# Unique Presentation and Management Approach of Pleural Solitary Fibrous Tumor

**DOI:** 10.1155/2019/9706825

**Published:** 2019-12-05

**Authors:** Tarek Aridi, Ayman Tawil, Mohamad Hashem, Joe Khoury, Roy A. Raad, Pierre Youssef

**Affiliations:** ^1^Faculty of Medicine, American University of Beirut, Beirut, Lebanon; ^2^Department of Pathology and Laboratory Medicine, American University of Beirut Medical Center, Beirut, Lebanon; ^3^Department of Surgery, Rafik Hariri University Hospital, Beirut, Lebanon; ^4^Department of Diagnostic Imaging and Interventional Therapeutics, Lebanese American University Medical Center-Rizk Hospital, Beirut, Lebanon; ^5^Department of Interventional Cardiology and Radiology, Hopital Libanais Geitaoui, Beirut, Lebanon; ^6^Department of Medical Imaging, Saint George Hospital University Medical Center, Beirut, Lebanon; ^7^Department of Surgery, Saint George Hospital University Medical Center, Beirut, Lebanon

## Abstract

Solitary fibrous tumor (SFT) of the pleura is an uncommon tumor that is often discovered incidentally on a routine chest X-ray. We report a case of a young female with a large, sessile, hypervascularized SFT of the pleura presenting with cardiopulmonary shock to a rural hospital with limited therapeutic interventions. We propose, in this case report, a unique multidisciplinary approach for the management of such a critical patient and the safe resection of the tumor.

## 1. Introduction

Solitary fibrous tumor (SFT), initially identified in the pleura, is a rare neoplasm that also arises in many extrapleural sites [1]. Pleural SFTs are mesenchymal tumors originating from either visceral or parietal pleura and can be localized or diffuse, pedunculated or sessile [2]. They constitute 5% of all pleural neoplasms, where 5-25% of them are malignant [1, 3]. We present the unique lifesaving management of a large sessile SFT of the left parietal pleura in a patient presenting with cardiopulmonary shock.

## 2. Case Report

A 44-year-old obese refugee female, with an unclear past medical and surgical history, presented to the emergency department of a rural private hospital with severe dyspnea and chest pain. The patient reported progressively increasing chest pain and cough in the prior three months, with a remarkable deterioration three days prior to admission. Chest radiograph showed complete opacification of the left hemithorax with labs illustrating a significant drop in hematocrit.

The patient deteriorated acutely with respiratory distress and was intubated. A bedside echocardiography showed mediastinal compression with a left-sided large pleural effusion. A chest tube was inserted draining 800 cc of dark blood. The patient's systolic blood pressure was undetectable at that moment. The patient went into a cardiopulmonary shock as a result of the severe hemorrhage.

**Figure 1:**
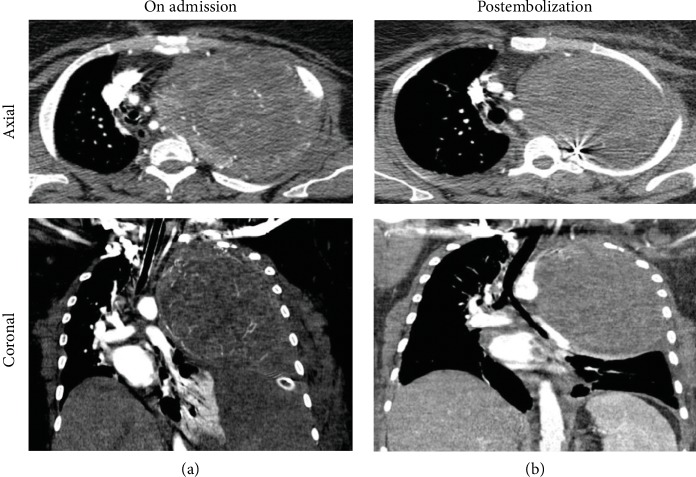
CT angiography of chest: (a) a large 14 × 12 × 8 cm mass in the left upper mid hemithorax with the left lung collapse; (b) markedly reduced vascularization postembolization.

After stabilization of the patient, a chest CT angiography showed a large 14 × 12 × 8 cm hypervascularized mass in the apex of the left hemithorax occupying more than two-thirds of the thoracic cavity with near total collapse of the left lung. In addition, large blood clots were seen consisting with left hemithorax, with secondary contralateral mediastinal shift (Figure 1(a)).

Due to financial and technical limitations in the rural hospital, the patient had to be stabilized prior to transfer to a university hospital for embolization. The decision was made to proceed immediately with a thoracoscopy to evacuate the clots and obtain biopsies. A bulky dark purple mass in the apex of the thorax was visualized arising from the parietal pleura with total collapse of the left lung. After obtaining the biopsies, two liters of clotted blood were evacuated resulting in the clearing of the left costophrenic angle on chest X-ray. The bleeding stopped spontaneously after clot evacuation requiring no additional surgical intervention. It is worth noting that there were no vascularized adhesions besides the primary tumor. Hence, the source of the hemithorax was attributed primarily to the bleeding mass.

**Figure 2:**
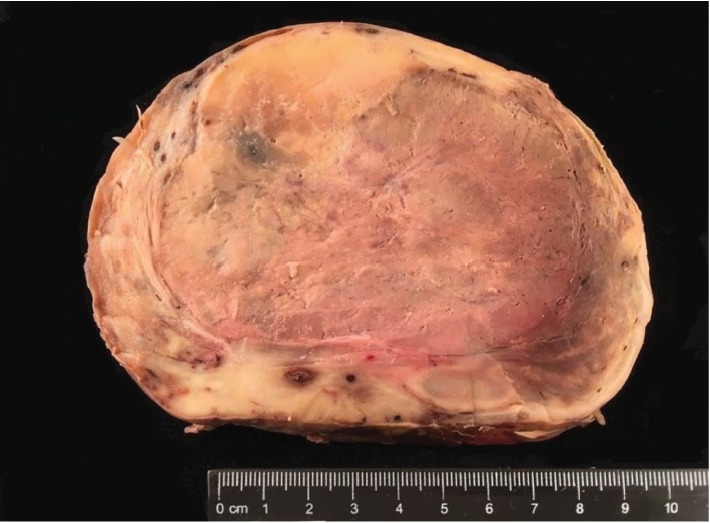
Gross appearance of the resected tumor: a large well-circumscribed tumor with firm fibrous cut surface.

A dramatic improvement in hemodynamic and respiratory parameters was observed allowing the intubated patient to be transferred the next day to a university hospital and undergo embolization. After extubation, repeat chest CT angiography showed marked reduction in tumor vascularization (Figure 1(b)). Under general anesthesia, a left posterolateral thoracotomy was performed in combination with the removal of the second, third, and fourth ribs. In addition, a thoracoscope was inserted, in a rendezvous technique, allowing better visualization of the apex and control of the left subclavian artery that was providing the major source of the mass' vascularization. The mass was resected with an estimated blood loss of 500 cc (Figure 2). The postoperative course was uneventful, and the patient was discharged on day 7 with significant amelioration in the left lung. On one-year follow-up, the patient showed no sign of recurrence.

## 3. Comment

Pleural solitary fibrous tumors are rare tumors with an incidence of 2.8 cases per 100,000 hospitalized patients annually [4]. SFTs are mainly asymptomatic but can present also with cough, chest pain, and dyspnea in 64%, hypertrophic osteoarthropathy in 20%, and hypoglycemia in 4% of the cases [5].

**Figure 3:**
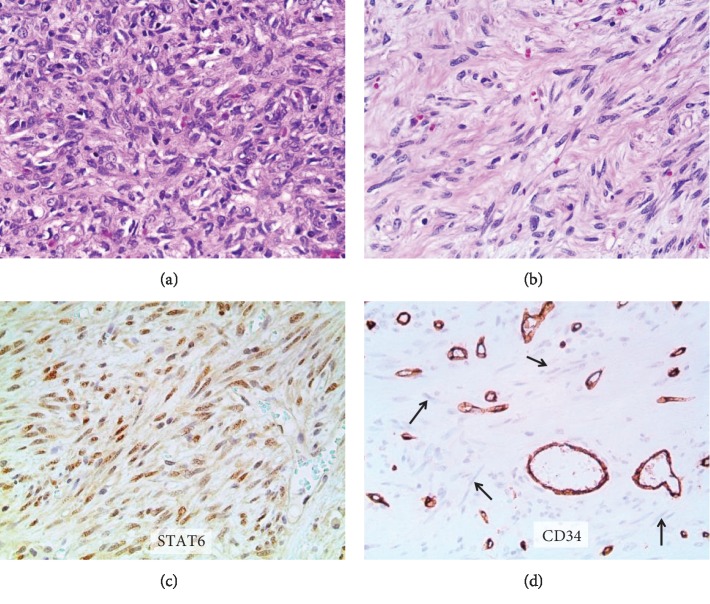
Histologic appearance of the tumor: (a) cellular area of the tumor uniform showing spindled cells with a patternless arrangement in a collagenous stroma (H&E, 200x); (b) less cellular area (H&E, 200x); (c) diffuse nuclear positivity for STAT6 (200x); and (d) CD34 highlighting the vascular channels but not the tumor cells (arrows) (200x).

The tumor consisted of a large (14 × 13 × 8 cm, 1005 g) well-circumscribed mass with a firm nonbulging cut surface. Histologically, it was composed of spindle-shaped fibroblast-like cells with a patternless architecture set in a collagenous stroma. Tumor cells showed no significant atypia and no detectable mitotic activity. The tumor also contained a rich network of thin-walled branching vascular channels. The histological features were those of a solitary fibrous tumor (Figures 3(a) and 3(b)). This was confirmed by diffuse nuclear staining for STAT6 (Figure 3(c)). One peculiar feature in this case is the lack of CD34 expression, a marker that is usually positive in SFTs (Figure 3(d)). Most SFTs pursue a benign clinical course; however, some can metastasize. The risk of metastasis is based on multiple criteria including tumor size, infiltrative margins, nuclear pleomorphism, tumor necrosis, and a high mitotic count (with the latter being the single most reliable criterion) [3]. The tumor in this case falls in the low risk category despite its large size.

Resectability of the tumor is a major determinant of clinical prognosis in SFT [6]. It is influenced by the mode of attachment, vascularization, and accessibility of the mass. SFTs are more commonly attached to the visceral pleura with the majority being pedunculated. The pedicles attaching those tumors are rich in feeding vessels [6]. Hence, an angiography should be performed to identify the pedicle [5]. Here, the preoperative embolization becomes necessary. Other alternatives include a mini-thoracotomy or thoracoscopic ligation of the vessels [5]. In contrast, tumors originating from the parietal pleura are more likely to be sessile [2]; thus, their vasculature cannot be controlled by simple ligation. Identifying the feeding vessels by angiography and interrupting blood flow by embolization has been shown to be the most effective in reducing intraoperative bleeding [7]. This is especially important in cases with multiple feeding vessels that are not confined to one easily accessible pedicle. Cardiopulmonary bypass and total circulatory arrest techniques have failed in controlling bleeding and thus do not serve as alternatives to embolization [8]. To better access the mass in a constricted area, the use of a thoracoscope in conjunction with the thoracotomy allows, in our opinion, a better visualization of the tumor.

It is important to always consider the accessibility and availability of therapeutic approaches when deciding on a management protocol. In rural hospitals, with limited medical equipment and financial means, it is necessary to adjust standard protocols to ensure the stabilization of the patient prior to proceeding with the transfer to university hospitals where more advanced techniques are available. Hence, as in our case, deciding on a diagnostic and therapeutic thoracoscopy prior to transferring the patient for embolization was a life-saving necessary step.

In conclusion, when suspecting a large, sessile, hypervascularized SFT with intrathoracic bleeding in rural areas with limited medical supplies, we recommend starting with a diagnostic and therapeutic thoracoscopy. Subsequently, the patient must undergo embolization followed by thoracotomy. Our case emphasizes the importance of the latter multidisciplinary approach for the management of large solitary fibrous tumors in patients presenting with a cardiopulmonary shock and in the presence of limited therapeutic techniques.

## References

[1] Vallat-Decouvelaere A. V., Dry S. M., Fletcher C. D. (1998). Atypical and malignant solitary fibrous tumors in extrathoracic locations: evidence of their comparability to intra-thoracic tumors. *The American Journal of Surgical Pathology*.

[2] Carretta A., Bandiera A., Melloni G. (2006). Solitary fibrous tumors of the pleura: immunohistochemical analysis and evaluation of prognostic factors after surgical treatment. *Journal of Surgical Oncology*.

[3] Demicco E. G., Wagner M. J., Maki R. G. (2017). Risk assessment in solitary fibrous tumors: validation and refinement of a risk stratification model. *Modern Pathology*.

[4] Okike N., Bernatz P. E., Woolner L. B. (1978). Localized mesothelioma of the pleura: benign and malignant variants. *The Journal of Thoracic and Cardiovascular Surgery*.

[5] Song J. Y., Kim K. H., Kuh J. H., Kim T. E., Kim J. H. (2018). Two-stage surgical treatment of a giant solitary fibrous tumor occupying the thoracic cavity. *The Korean Journal of Thoracic and Cardiovascular Surgery*.

[6] England D. M., Hochholzer L., McCarthy M. J. (1989). Localized benign and malignant fibrous tumors of the pleura. A clinicopathologic review of 223 cases. *The American Journal of Surgical Pathology*.

[7] Weiss B., Horton D. A. (2002). Preoperative embolization of a massive solitary fibrous tumor of the pleura. *The Annals of Thoracic Surgery*.

[8] Aydemir B., Çelik S., Okay T., Doğusoy I. (2013). Intrathoracic giant solitary fibrous tumor. *American Journal of Case Reports*.

